# An Unusual Cause of Pseudomedian Nerve Palsy

**DOI:** 10.1155/2011/474271

**Published:** 2011-10-12

**Authors:** Zina-Mary Manjaly, Andreas R. Luft, Hakan Sarikaya

**Affiliations:** Department of Neurology, University Hospital Zurich, Frauenklinikstraße 26, 8091 Zürich, Switzerland

## Abstract

We describe a patient who presented with an acute paresis of her distal right hand suggesting a peripheral median nerve lesion. However, on clinical examination a peripheral origin could not be verified, prompting further investigation. Diffusion-weighted magnetic resonance imaging revealed an acute ischaemic lesion in the hand knob area of the motor cortex. Isolated hand palsy in association with cerebral infarction has been reported occasionally. However, previously reported cases presented predominantly as ulnar or radial palsy. In this case report, we present a rather rare finding of an acute cerebral infarction mimicking median never palsy.

## 1. Case

A 60-year-old woman presented to the emergency department with difficulty in moving the thumb, index, and middle finger of her right hand. She had noticed the symptoms in the morning on waking up after an uneventful night without any preceding intake of alcohol or hypnotics. She felt no sensory disturbances. At the time of presentation, she was being treated for arterial hypertension, osteoporosis, and depression.

**Figure 1:**
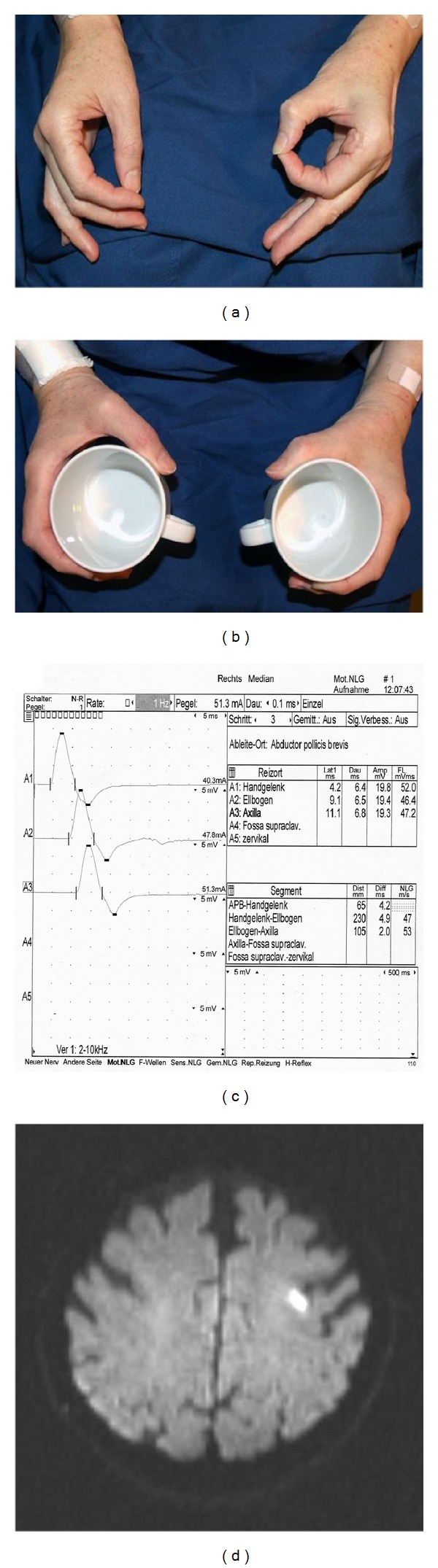
(a) Pathological “circle sign” of the right hand. The patient is unable to correctly oppose the tips of digits I and II due to weakness of flexor pollicis longus muscle and the flexor digitorum profundus muscle of the index finger, (b) Lüthy's bottle sign. See the gap between the cup and the skin web due to weakness of thumb adduction, opposition, and flexion in median nerve lesion, (c) Normal neurography of the right median nerve, (d) Diffusion-weighted magnetic resonance imaging showing an acute infarction in the “hand knob” area of the left precentral gyrus.

Clinical examination of the right hand revealed a moderate paresis (strength 3-4 on the Medical Research Council (MRC) scale [1] for muscle strength) of Mm. flexor digitorum longus et brevis I-III and M. flexor carpi radialis, with prominent impairment of abduction and opposition of the thumb (M3) (Figures 1(a) and 1(b)). Ulnar and dorsal wrist flexion as well as adduction and abduction of fingers II-V was normal. Similarly, proximal arm muscles, somatosensory perception, muscle tone, and tendon reflexes were unaffected. Further clinical examination, including function of language, cranial nerves, and plantar reflex, did not show any abnormalities. 

Although the paretic muscles were innervated by the median nerve exclusively and thus suggested a peripheral nerve lesion, the findings were not compatible with a precise localization in the anatomic course of the nerve. Therefore, we subsequently performed a neurography of the right median nerve, which was normal (Figure 1(c)). Magnetic resonance imaging (MRI) on the same day revealed a small diffusion restriction in a part of the left precentral gyrus that is known as “the hand knob” area (Figure 1(d)) [2].

## 2. Discussion

This case underlines the value of precise neurological examination in patients presenting with focal limb paresis and demonstrates that ischemic stroke can mimic a peripheral nerve lesion. In our patient, all affected muscles were innervated by the median nerve, while muscle groups innervated by the radial and ulnar nerve were normal. There were no prominent signs suggesting a central origin of the symptoms as muscle tone and cranial nerves were normal, tendon reflexes symmetric, plantar reflex normal, and higher cortical functions unaffected. 

Classical causes of nontraumatic median neuropathy in the forearm include carpal tunnel syndrome (CTS), the anterior interosseus syndrome (also known as Kiloh-Nevin syndrome) and the pronator teres syndrome. The carpal tunnel is by far the most frequent site for peripheral nerve entrapment with high prevalence in older women [3]. However, the lack of sensory symptoms such as (nocturnal) paraesthesia and the acute symptom onset in our patient were not compatible with CTS. The anterior interosseus syndrome is a less frequent cause of median nerve palsy and occurs spontaneously or results from an isolated traumatic lesion of the anterior interosseus nerve, a pure motor branch of the median nerve [4]. The lesion presents with paresis of the long finger flexors I-III and is characterized by pathological “circle sign” (see Figure 1(a)). The pure motor paresis in our case matched this syndrome well, but the affection of M. flexor carpi radialis suggested a more proximal lesion. Repeated pronation-supination movements of the forearm (e.g., after extended screwdriver use) can be the cause of the pronator teres syndrome with compression of the median nerve between the two parts of the pronator teres muscle [5]. Our patient, however, neither had a typical history for such, nor did she present classical findings, that is, tenderness over the proximal median nerve aggravated by resisted pronation. Finally, compression of median nerve can also occur in “paralysie des amants”, when the partner's head lies in the cubital fossa over night. In this case, there was no history suggesting this cause.

In summary, the results of the neurological examination were not compatible with a peripheral localization and thus prompted us to consider a central lesion. Normal neurography was helpful to quickly disregard a peripheral lesion and to consider MRI, which demonstrated cerebral infarction in the so-called hand knob area of the left precentral gyrus.

Isolated hand palsy in association with cerebral infarction has been occasionally reported. Most of these patients had either a fall-hand mimicking radial nerve palsy or predominant involvement of fingers innervated by the ulnar nerve [6, 7]. In contrast, our patient had no weakness of ulnar-side fingers. To our knowledge, “hand knob” infarction with selective impairment of median nerve innervated muscles is a very rare finding [8]. The etiology of focal cortical infarction is mainly thromboembolic due to large artery atheromatosis [9], as this was the case in our patient. Overall, prognosis seems to be favorable. In line with this, our patient regained normal muscle strength at the third day after stroke onset. Identification of “pseudoperipheral palsies” is crucial to immediately introduce appropriate diagnostic therapeutic measures for prevention of a recurrent ischemic stroke.
